# Lack of association of statin use with vitamin D levels in a hospital based population of type 2 diabetes mellitus patients

**DOI:** 10.12669/pjms.341.11977

**Published:** 2018

**Authors:** Khalida Iqbal, Najmul Islam, Iqbal Azam, Naseema Mehboobali, Mohammad Perwaiz Iqbal

**Affiliations:** 1 Khalida Iqbal, Department of Biological & Biomedical Sciences, Aga Khan University, Karachi, Pakistan; 2 Najmul Islam, Department of Medicine, Aga Khan University, Karachi, Pakistan; 3 Iqbal Azam, Department of Community Health Sciences, Aga Khan University, Karachi, Pakistan; 4 Naseema Mehboobali, Department of Biological & Biomedical Sciences, Aga Khan University, Karachi, Pakistan; 5 Mohammad Perwaiz Iqbal, Department of Biological & Biomedical Sciences, Aga Khan University, Karachi, Pakistan

**Keywords:** Diabetes mellitus, Pakistani population, Statins, Vitamin D levels

## Abstract

**Objective:**

To investigate the relationship of statins (drug given to reduce serum levels of LDL-cholesterol) on vitamin D levels of Pakistani type 2 diabetes mellitus (DM) patients in a hospital in Karachi.

**Methods:**

In a cross-sectional survey, 312 consecutive patients with type 2 DM (219 males and 93 females, age 22-70 years) were recruited with informed consent. A questionnaire was administered to find out whether they were statin users or non-users. Serum was analyzed for concentrations of 25(OH) vitamin D [25(OH)D] and other related biomarkers such as serum cholesterol, triglycerides, HDL-cholesterol, LDL-cholesterol, phosphate and calcium using kit methods. Multiple Linear Regression was used to evaluate association of statin use with serum levels of vitamin D while adjusting for related covariates including duration of statin use, duration of type 2 DM and smoking.

**Results:**

Mean concentrations of serum cholesterol, and LDL-cholesterol were lower among statin users compared to statin non-users (*P* < 0.01), while HDL-cholesterol levels were higher (*P*<0.01). No relationship was observed between statin use and serum levels of vitamin D (*P*=0.768), when adjusted for age, gender, BMI, duration of type 2 DM, smoking, serum cholesterol and LDL-cholesterol. The adjusted regression coefficient (β) and standard error [SE(β)] for statin use duration were 0.012 (0.042), when serum levels of vitamin D was taken as an outcome.

**Conclusion:**

Lack of association was found between statin use and vitamin D levels in a hospital-based population of Pakistani patients with type 2 DM.

## INTRODUCTION

Statins are a group of cholesterol lowering drugs which inhibit HMG-CoA reductase, a key enzyme in the cholesterol biosynthesis. They have a well-established role in decreasing serum concentration of low density lipoprotein (LDL) cholesterol, thereby reducing the risk of atherosclerotic cardiovascular disease (CVD).^1^ A Cochrane review in 2013 has indicated a role of statins in the primary prevention of CVD in people with no history of this disease.^2^ An association between CVD risk and vitamin D deficiency has also been reported in a number of studies.^3^,^4^

The deficiency of vitamin D is a global health problem and its prevalence is very high in South Asian populations including Pakistan.^5^,^6^ A few studies have shown that statins significantly increase serum levels of vitamin D metabolite, 25-hydroxyvitamin D (25(OH)D).^7^,^8^ However, a recent systematic review and meta-analysis of 7 studies including 5 randomized clinical trials showed no significant effect of statin treatment on plasma levels of vitamin D.^9^ It has even been suggested that statin therapy may impair vitamin D status, thereby leading to increased risk of CVD.^10^-^11^ More recently, a meta-analysis on the effect of statins on vitamin D levels was inconclusive and reported “conflicting directions of effects from interventional and observation studies”.^12^

Since no studies have been carried out on South Asian population on the relationship of statin use and vitamin D status, we embarked on investigating this relationship in Pakistani diabetic patients who are often put on statins to reduce CVD risk while managing the blood glucose levels. Therefore, the objective of this study was to find out if there is any association between statin use and vitamin D levels in a hospital based population of Pakistani patients with type 2 diabetes mellitus (DM).

## METHODS

In a cross-sectional survey for the assessment of the role of statins on vitamin D levels in type 2 DM patients, 312 consecutive patients (age 22-70 years; 219 males and 93 females) were recruited from the Endocrinology Clinics of the Aga Khan University Hospital (AKUH) with informed consent. These patients had confirmed diagnosis of type 2 DM on the basis of clinical history and fasting serum glucose > 126 mg/dl as per guidelines of the International Diabetes Federation. According to the inclusion criteria, they had not been taking vitamin D supplements for the last 6 months and were not suffering from any of the chronic diseases such as tuberculosis, liver disease, uremia or cancer and were not pregnant. Majority of the recruited patients (65%) were newly diagnosed diabetes patients with duration of diabetes less than 6 months. Seventeen percent of patients had duration of the disease for more than 60 months. All the patients were on oral hypoglycemic drugs. Those on statins had been on daily dosages 5 mg (13%), 10 mg (55%) and 20 mg (32%). The study had been approved by the Ethics Review Committee of the Aga Khan University.

A brief questionnaire was used for demographic information and to find out whether they were using statins or not. Ten ml fasting (at least 4 hours) blood was collected and serum/plasma was analyzed for 25(OH) vitamin D [25(OH)D], cholesterol, LDL-cholesterol, HDL-cholesterol, triglycerides, phosphate and calcium using kit methods (Roche Diagnostics Indianapolis, IN). For quality assurance, standard controls with high and low concentrations of biomarkers were run along with the test samples.

### Statistical analysis

Statistical analyses were carried out using Statistical Package for Social Sciences® (SPSS) software version 19 for Windows® (Apache Software Foundation, USA). Mean values of continuous variables such as age, BMI, serum levels of cholesterol, triglyceride, HDL-cholesterol, LDL-cholesterol, phosphate, calcium, 25(OH)D, duration of diabetes and duration of statin use were expressed as mean±SD, while categorical variables such as gender and smoking status were expressed as n(%). Mean values of variables in two groups (statin users and statin non-users) were compared using Independent sample t test. Multiple linear regression was used to observe the relationship between vitamin D levels and statin use, which was also adjusted for demographic and health related covariates.

## RESULTS

As shown in Table I, mean age and duration of diabetes in the statin non-users group were significantly lower compared to the statin users group (*P* < 0.01). Similarly, mean concentrations of serum cholesterol and LDL cholesterol were significantly lower in the statin users group compared to the non-users group (*P* < 0. 01). However, HDL-cholesterol levels were significantly higher in the statin users group (*P* < 0.01). No significant difference was found when the mean values of 25(OH)D between statin-users and statin non-users were compared using Independent sample t test (*P* = 0.23). No significant differences with respect to other variables were found between the two groups.

**Table-I:** Demographic and clinical characteristics of type 2 DM patients with respect to statin use and non-use.

Variable	Statin nonusers (n=160)	Mean±SD	Statin users (n=152)	Mean±SD	P*
	
	n(%)		n(%)		
*Gender*					
Males (n=219)	108(67.5)	-	111(73)	-	0.29
Females (n=93)	52(32.5)	-	41(27)	-	
*Smoker (current)*					
No	128(80)	-	122(80.3)	-	0.95
Yes	32(20)	-	30(19.7)	-	
Duration of DM (months)	-	35.8±46.8	-	63.9±70.3	<0.01
Age (yr)	-	45.6±10.5	-	50.8±8.7	<.01
BMI (kg/m^2^)	-	29.8±5.5	-	29.9±5.2	0.22
Serum cholesterol (mg/dl)	-	162±48	-	127±46	<0.01
Triglyceride (mg/dl)	-	160±86	-	144±72	0.08
HDL-cholesterol (mg/dl)	-	25.03±7.80	-	29.29±8.91	<0.01
LDL-cholesterol (mg/dl)	-	90±36	-	63±39	<0.01
Phosphate (mg/dl)	-	3.6±0.70	-	3.75±0.74	0.27
Calcium (mg/dl)	-	9.93±1.8	-	9.71±1.93	0.30
25(OH)D (ng/ml)	-	18.62±11.63	-	20.3±11.6	0.23

* *P* was obtained by comparing mean values of two groups (statin users and non-users) using Independent samples t test, while percentages in the two groups (statin users and non-users) were compared using chi square.

Regarding mean duration of statin use and dosages of statins, out of 152 statin users, the data of 118 were available for regression analysis. Mean duration of statin treatment among the statin users group was 17.99±21.42 months, while the mean dosage of statins in this group was 12.7±5.3 mg.

The effect of different factors, particularly duration of statin use is shown in Table II. Gender, age, BMI, duration of DM, levels of serum cholesterol, HDL-cholesterol and calcium were found to be significant at the univariate level.

**Table-II:** Effect of statin use on the serum levels of vitamin D using multiple linear regression.

Variable	Crude β[SE(β)]**	P	Adjusted* β[SE(β)]**	P
Statin use duration (months)	0.07(0.039)	0.076	0.012(0.042)	0.768
Age (yr)	0.227(0.067)	0.001	0.117(0.071)	0.099
*Gender*		-		0.018
Male (Ref.)***	-			-
Female	3.176(1.47)	0.031	3.496(1.47)	-
BMI (kg/m^2^)	-0.338(0.133)	0.005	-0.294(0.132)	0.027
Type 2 DM duration (months)	0.041(0.011)	<0.001	0.035(0.013)	0.006
*Current Smoking*	-	0.388	-	0.582
No (Ref.)***	-	-	-	-
Yes	1.495(1.728)	-	0.974(1.765)	-
Cholesterol (mg/dl)	-0.28(0.014)	0.043	-0.057(0.019)	0.003
LDL-C (mg/dl)	-0.011(0.017)	0.500	0.062(0.024)	0.01
HDL-C (mg/dl)	0.16(0.076)	0.035	-	-
Triglyceride (mg/dl)	0.006(0.008)	0.468	-	-
Phosphate (mg/dl)	-0.455(0.913)	0.618	-	-
Calcium (mg/dl)	-0.714(0.353)	0.044	-	-
Constant	-	-	22.915(5.740)	<0.001

* Model was adjusted for age, gender, BMI, duration of type 2 diabetes mellitus, current smoking, serum cholesterol and LDL-cholesterol with coefficient of determination (R^2^)= 0.135.

** β[SE(β)] refers to regression co-efficient with its standard error.

*** Ref. indicates the reference group category in regression.

At multivariable level, when the relationship between duration of statin use and serum level of vitamin D was adjusted for other factors, which were either biologically important or significant at the univariate level, the variables like gender, BMI, duration of type 2 diabetes, serum levels of cholesterol and LDL-cholesterol were found to be significantly associated.

Current smoking was kept in the model because of its biological importance.^6^ Regarding the duration of statin use, one month increase in statin use was found to be responsible for an average increase in vitamin D level by 0.012 ng/ml when adjusted for age, gender, BMI, duration of type 2 DM, current smoking, serum cholesterol and LDL-cholesterol. The value of coefficient of determinations (R^2^) of 0.135 indicates that 13.5% variation in serum level of vitamin D is explained by the investigated covariates.

## DISCUSSION

There are numerous reports about high prevalence of hypovitominosis D in South Asian general population as well as patients with type 2 DM.^13^-^15^ Since diabetic patients have an additional risk of developing CVD, they are often prescribed statins to control their LDL cholesterol along with usual management of their serum glucose level. However, long-term use of statins may produce hepatotoxicity. Since cholesterol metabolism and vitamin D both share a common metabolic pathway, it is possible that prolonged use of statins would not only inhibit cholesterol biosynthesis but also reduce levels of 7-dehydrocholesterol, a precursor molecule for vitamin D3, thereby leading to low concentration of vitamin D in the body.^16^ This would render such patients vulnerable to risks associated with vitamin D deficiency. However, in the present study we found no difference in serum levels of vitamin D in statin users and non-users in type 2 DM patients. Similar findings have been reported by Ertugrul et al, who have shown no significant effect of pravastatin and fluvastatin on serum levels of 25(OH)D.^17^ In a randomized controlled trial on healthy postmenopausal women, simvastatin was found to have no effect on vitamin D status even after 52 weeks of treatment.^18^ Similar results have been found by Mazidi et al on an Iranian population of patients with dyslipidemia.^19^ On the contrary, Sathyapalan et al have reported increased concentration of 25(OH)D following therapy with atorvastatin in type 2 DM patients in UK.^20^ Similarly, 4 studies (two carried out in Turkey, one in Spain and another one in Germany) have also shown increased serum levels of 25(OH)D following treatment with rosuvastatin and atorvastatin.^8^,^17^,^21^,^22^ Most of these studies reporting increased levels of 25(OH)D following statin treatment had small sample sizes which could lead to overestimation of therapy effect, and therefore, it was difficult to derive any substantive conclusion. Perhaps, this could be the reason that most recent studies on this pleotropic effect of statins have failed to show any significant increase in vitamin D levels.^19^,^23^ A recent meta-analysis of data from seven studies failed to show any significant effect of statins treatment on plasma concentrations of vitamin D.^9^ All these reports lend support to our current results indicating lack of association between statin use and serum levels of vitamin D in type 2 DM patients. The multiple linear regression model to investigate this relationship while adjusting for other related factors mentioned above is a strength of this study.

Our results must be seen in the context of certain limitations. Our collection of blood samples was across the whole year and the samples could not be divided into those collected during winter, spring or summer. Furthermore, no dietary information was obtained. However, in spite of these limitations, we have been able to report lack of association between statin use and vitamin D levels in a hospital-based population of type 2 DM patients using a reasonably large sample size.

In order to obtain conclusive evidence regarding the relationship of statin use with vitamin D status in South Asian population, prospective, randomized, placebo controlled and double blinded multicentric clinical trials with large sample size would be required.

## CONCLUSION

There is no association between statin use and levels of vitamin D in a hospital-based Pakistani population of type 2 DM patients.

### Authors' Contributions

**NI and MPI** conceived and designed the experiments.

**KI and NM** performed the experiments.

**IA, KI, NI, and MPI** analyzed and interpreted the data.

**KI, MPI, and NM** contributed reagents, materials and analysis tools.

**MPI, NI and IA** prepared the final manuscript.
